# The Dietetic Movement

**Published:** 1886-03

**Authors:** 


### HALL'S JOURNAL OF HEALTH

Vol. 33. MARCH, 1886. No. 3.

### THE DIETETIC MOVEMENT.

*The Journal of Reconstructives* has an article under this heading, which carries us back many years, and brings to mind men who thirty years ago were lecturing and telling the doctors the same thing now advanced as a novel movement. It is a curious fact, but one of universal application, that all reforms in the practice of medicine are bought about by those whom the profession point to as "cranks," and not wholly without reason, for they were many of them very peculiar, but they almost always had some ideas which were good and which have lived through all the various changes that have taken place since they made a stir by denouncing the quackery of the old practice. We remember when we were a boy, the Thompsonian practice, which was denounced in most bitter terms, as arrant quackery, by men who were wielding the lancet at every patient for all kinds of disease; who were dosing with opium, calomel and all kinds of drugs, which have since seen a wonderful diminution in their use. The old Thompsonian song said:

"Mercury, arsenic, opium, too,
"Physic, blisters, lance, adieu,
"And all who use them we defy,
"Excepting when we wish to die."

The men who used this doggerel were, as we have said, cranks; but they did some good, for they kept up the cry until doctors began to think, to investigate and to find out that their practice was not producing the good effects they had imagined. Thompsonianism has, so far as we know, gone out of the world, but it has left behind it some truths that are now universally acknowledged. And it is well said by the *Reconstructionist* that "there is, moreover, a growing skepticism as to the efficacy of many of the older remedies, a growing tendency to abandon the use of the more powerful drugs for specially prepared and easily assimilated pharmaceutical products."

This was told long ago, and there have been physicians for the last fifty or sixty years, who have been crying in the wilderness of medical ignorance, against the old, death dealing practice, which gave the patient much more to recover from than he would have, had he been left entirely alone. Think of the patient dying with fever solely for want of water, as many of them have; when medical ignorance condemned to death rather than to have one drop of good cold water, and think, too, how many recovered that got at the water in some way, yet the regular doctor would not learn. It was not until the water cure became known to the world, that water was allowed to their patients. Now all have it, because water has been demonstrated to be beneficial in fevers, and in many other diseases. In fact, it is now seen by nearly all the profession that they have learned much from these "cranks;" in fact, that these men have been the pioneers in the practice of many reformatory modes. The less medicine a doctor gives the greater is his success. It has long been known by our oldest and best physicians that it is only occasionally necessary to give medicine; that bread pills are much better for a variety of patients than anything stronger.

All men who have taken an interest in the practice of medicine, who have more interest in discovering the best manner of curing disease than in maintaining a profession, have been denounced as quacks by the regulars, who have drawn around them the shield of law, while others have forced them to the adoption of better modes of practice. The journal we have quoted, says: "The new departure which it is claimed is to form an epoch in medicine, if not to effect a revolution in medical practice, is told in three short words—*feed the patient.* All diseases tend to reduce, to weaken and wear out the system. The required medication often tends to still greater debility. We see, says Trousseau, alterative medicine hindering and destroying the action of plastic force, opposing the changes of living chemistry by weakening the nutritive properties of the blood and the tonicity of the solids."

Men who have kept their eyes open for the last forty years, and have taken whatever appeared as an improvement from whatever source it came, whether from an old professional or from some "quack" have taught the profession many lessons which they at last propose to look at and adopt. It is not a new thing, by a great deal, to men who have been looking rationally at the practice of medicine, as they haev at other matters of great interest to humanity. We quote farther from the *Journal of Reconstructives.*

"Instead of dwelling at great length on cathartics, alteratives and expectorants, an opportunity will be afforded the professor to discourse eloquently on aliments, haematics and reconstituents. Instead of the headings Therapeutics and Materia Medica in the the text-books, the student will read Dietetics, Therapeutics and

Materia Medica. 'Dietetics,' says Dr. Roberts in a recent address, 'covers more ground than any other branch of the healing art.' Nothing can be truer. The 1,126 diseases of the nomenclature all have one thing in common, they all reduce the patient—so do many of their remedies. Any practice which counteracts this tendency must certainly cover more ground than any other branch of the healing art.

"Here is a field of operation! an endless opportunity for experiment, a scope for investigation and discovery unlimited. Why should not each of the 1,126 diseases have its appropriate counteracting food as it now has its counteracting remedy? Why should we not have manuals of alimentary therapeutics as well as manuals of alterative therapeutics? Why may we not have diet-rolls for other diseases than gout and diabetes, other food specifics besides cod-liver oil for phthisis? Why may not the initial attempt at the classification of foods as flesh formers, combustibles and mineral producers be pushed on till a true science is created and we have foods differentiated for membrane and muscle, for nerve and for parenchyme? Why may we not have a Materia Dietetica in which the student shall learn what he can build up or restore with aliments or reconstituents, as he has already learned, often too well, what he can combat and drive out with alteratives, evacuants and the nervina depressoria."

"It is believed that the present tendency among our best physicians largely to substitute food instead of medicine in the treatment of disease is not to be looked upon as a mere fashion like hydropathy, the hot-water treatment, and other similar ephemeral movements. The dietetic movement may certainly be said to have a wider and deeper meaning. 'The effects of diet,' says Dr. Roberts, 'are profound and far-reaching and cannot be over-estimated.' Indeed, the present indications are that a change in the relations of food and medicine is slowly taking place simultaneously along with changes in the relations of physiology to pathology. Just as pathology seems to be turning into physiology, so therapeutics seem inclined to undergo transformation in the direction of dietetics."

"For something more than a generation, ever since the present century, inquiry into the origin and workings of cell life has begun to bear fruit, the conscience of the physician has been growing tender. The usual remedies are often of a nature injurious to the organism. We have heard repeated the wise and witty remark of Dr. Holmes that if all the apothecaries' bottles were emptied into the sea, it would be all the better for the men and all the worse for the fishes."

We can say also that we heard one of the best physicians in the

State of Pennsylvania say in a lecture: "If we should hear, on our awaking to-morrow morning, that every physician in the world was dead, the world would be the better for it!" He then said that he knew many most excellent physicians—men whom he would readily call were he sick—but there was such a vast majority of miserable men who were doctors because they wanted to do something for a living, that the world would be immensely better if it were rid of them. And yet this profession wants all the force of special legislation to keep their faculty in a class that shall drive out all who do not belong to them. It may succeed for a time, but is just as sure to ultimately fail as the world is to continue to move.

